# Animal Calorimetry

**Published:** 1915-02

**Authors:** 


**Animal Calorimetry.** Graham Lusk, Cornell Medical School, "Johns Hopkins Hosp. Bull.," January, 1915. This article is not susceptible of abstract but we cull the following list of amino-acids—9 of the 17 of which proteid may be composed—that are more or less converted into glucose and eliminated in the urine in diabetes: Alanin and glycocoll completely, aspartic and glutamic acid partly, serin, cystin, ornithin, prolin and arginin not specified.

